# Effectiveness of teachers' direct and indirect written corrective feedback provision strategies on enhancing students’ writing achievement: Ethiopian university entrants in focus

**DOI:** 10.1016/j.heliyon.2024.e24279

**Published:** 2024-01-11

**Authors:** Baymot Mekuriaw Wondim, Kassie Shifere Bishaw, Yinager Teklesellassie Zeleke

**Affiliations:** Department of English Language and Literature, Faculty of Humanities, Bahir Dar University, Bair Dar, Ethiopia

**Keywords:** *C*orrective feedback, Written corrective feedback, Sustained feedback, Writing achievement, University entrants, Ethiopia

## Abstract

Written corrective feedback (hereafter WCF) has gained great emphasis from a considerable number of studies in second language (L2) writing history; however, an increasing number of previous studies have stressed its importance in helping learners develop their L2 writing abilities there are unresolved controversies regarding the significance and efficacy of various forms of written corrective feedback. Thus, this study was initiated to see the effects of teachers' written corrective feedback on university-entrant students' English language writing achievement. A quasi-experimental research design involving a test as a data-gathering tool was used. To that end, three intact freshman classes were selected and assigned into two experimental groups and one comparison group from a university in northwest Ethiopia. Test scores from self-descriptive paragraph writing were analyzed using a one-way ANCOVA, and the results showed that WCF has an influential role in improving university entrat learners' writing performance. Therefore, the findings from the study showed that both experimental groups performed better than the participants in the control group, indicating that both WCF provision strategies play vital roles in enhancing English language learners' writing performance as university entrants. Nevertheless, the study's findings revealed that statistically significant improvements in the writing proficiency of the study participants in both experimental groups were found the results confirmed that learners who received direct WCF along with metalinguistic explanations performed better than their peers in the indirect group who received indirect WCF. Consequently, it can be concluded that both direct and indirect WCF are crucial pedagogical strategies in improving learners' writing abilities, even though it was still found that direct WCF with a metalinguistic explanation was the most effective strategy in assisting EFL university entrants in improving their writing skills.

## 1 Introduction

Writing is a skill that allows individuals to produce insightful, indisputable documentation of their notions, thoughts, and spirits; as such, writing proficiency has become recognized in contemporary society as a social and intellectual asset. The importance of writing is emphasized in great detail in the works of Chappell (2011) who claimed that writing can be used to communicate one's personality, improve communication, sharpen one's thinking abilities, construct coherent and convincing arguments, offer and receive feedback, help one reflect on and reevaluate ideas later on, and get ready for education and the workforce as cited in Klimova [1]. In light of this, when we consider every course involves ones ability to write, writing skills are appeared to be very important as indicated by Junianti, Pratolo and Wulandari [2]; thus, it can be claimed that writing is one of the most significant language skills as it is widely utilized in every aspects of one's lives; for instance, writing plays irreplaceable in both both the workplaces and higher educations. Several scholars said that writing gives students lots of chances to look for contemporary approaches to communicate their ideas and thoughts in a foreign language [3]. Effective writing in the English language is especially important for learners' academic achievement in Ethiopia, the study's context, where the English language is used as the medium of instruction for other content subjects beginning in grade seven and continuing through higher education. This is due to the fact that writing is one of the macro skills that is essential to students' academic success as well as the development of other language proficiency areas for second language learners. Moreover, because writing involves a variety of terminology while writing, it helps students improve their grammar, vocabulary, and ability to read and listen well.

Although writing plays a crucial role in student's academic lives, learning to write is a complicated cognitive task requiring students to be proficient in various aspects of language. These aspects range from students' educational backgrounds and personal interests to linguistic, psychological, and cognitive phenomena [4]. Therefore, since writing involves complex cognitive processes to complete a final draft well, scholars believe that writing is one of the most advanced language abilities compared with other language abilities [5]. Thus, producing a cogent, fluent, and error-free piece of writing will probably be a constant challenge for EFL students [6]. Due to its challenging nature or other related variables, in the Ethiopian EFL context, it is common to observe that this skill is only addressed superficially due to several constraints that EFL teachers and students deal with. In line with this, several studies have shown that students in higher education in Ethiopia are weak in their writing skills; consequentially Ethiopian EFL University Students cannot articulate their thoughts clearly in writing as indicated in Mulgeta [7] and Surur and Dengela [8]. From this, it can be deduced that most of Ethiopian EFL first-year university students struggle when they are asked to write a simple sentence; due to these reasons, it is expected to see a lot of errors in their written text, and they are incapable of composing error-free simple sentences [7,8]. However, it is generally unacceptable and impractical to expect EFL students to produce flawless texts students at higher academic institutions will encounter challenges in their educational journey if they are not proficient writers, since most of evaluations in these institutions rely on written content. Although it is a very complex language skill, scholars like Han and Hiver [9] strongly warned that writing is a talent that can no longer be overlooked or addressed superficially.

The cognitivist perspective of errors guided by Chomsky's [10] and his associates' works views errors as learning signals so they cannot be avoided. In a similar vein, Corder [11] claimed that errors are indicators of progress in learning. Therefore, it was asserted that errors are inevitable variables in L2 classes in general and writing classes in particular, which encouraged researchers in applied linguistics and language teaching to look for possible strategies that may aid students in overcoming their errors and producing accurate written output [12]. However, L2 errors are viewed as indicators of progress in language learning; if they are not promptly and properly fixed, they will eventually become fossilized and used as proper language forms. Therefore, in this area of concern, scholars' recommend that corrective feedback (hereafter CF) due to it is both a practical remedy ideal approach for students' competence issues and a key element in improving learners' writing skills [13]. Although it was generally agreed that CF plays a crucial role in assisting L2 learners, this conclusion encountered intense opposition from several prominent scholars like Truscott [14], who is the most extremist scholar having a negative outlook on the role of CF in writing classrooms, and he vehemently recommended the elimination of its use. This scholar mainly linked CF as it is harmful and prevents students' interaction and the flow of ideas, so he advises teachers to avoid using CF in L2 writing sessions completely.

Error Analysis, on the other hand, was found to have a strong position in supporting corrective feedback as it is concerned with identifying and organizing remedial courses as well as creating relevant materials and teaching methodologies based on the results of theoretical error analysis by Erdogan [15], in this response, corrective feedback can be listed among the remedial ways for the identified learners' linguistic errors. Thus, the consensus is that CF is undeniably necessary to facilitate L2 knowledge because mistakes and errors are expected at all stages of learning; this calls for some practical solutions and, to this end, paradigm shifts and changes in perceptions about effective and meaningful ways to give students feedback are needed [16]. Similarly, scholars whose works are guided by sociocultural theories see the potential of corrective feedback in language acquisition and learning [[17], [18], [19]]. Therefore, over the last several decades, WCF has received a great attention in second language (L2) writing and L2 acquisition [20,21]. From this, it can be stated that the provision of WCF has clear and direct relevance for English language teachers since teachers can control over controlling students’ written accuracy [19]. Thus, it can be concluded that WCF is a helpful and essential pedagogical strategy for learners to overcome L2 errors in their writing, acquire accurate English, improve their grammatical precision over time, prevent the fossilization of improper language structures, and strengthen their writing abilities [22].

However, it was agreed that written corrective feedback is an important pedagogical element there are no conclusive and consistent results on the effectiveness of direct or indirect WCF. These ongoing and endless debates over the efficacy of WCF may be linked to several variables that previous studies employed in their research designs. For instance, Eslami [23] carried out a study on the effects of direct and indirect CF methods on EFL students’ writing, focusing on the simple past tense, and reported that the indirect feedback group performed better than the direct feedback group. In similar concern, Benson and DeKeyser [24] conducted a study on how WCF and language aptitude affected verb tense accuracy, focusing only on the simple past tense and the present perfect tense, and they found that participants with a high language attitude to tense and speakers with a low language attitude to tense preferred metalinguistic direct feedback. Likewise, Westmacott [25] compared direct and indirect WCF procedures and concluded that indirect error correction is more efficient than direct error correction strategies. On the other hand, more studies reported that direct written CF is the most effective provision strategy over its counterpart [26,27].

When it comes to the Ethiopian context, from the researchers' best reading, with the exceptions of a few studies by, for example, Wondim, Bishaw and Zeleke [28], who examined the effects of WCF on Ethiopian first-year university students' writing achievement, and reported that WCF has a potential impact on improving learners' writing achievement; however, they did not convincingly demonstrate whether indirect WCF helps EFL learners attain writing success or not. One of the study's major flaws was lack of a clear comparison between students in the treatment group who received direct WCF and a metalinguistic explanation with those in the comparison group who received no feedback. In these scholars study, the comparison of the students writing achievements in the indirect group (who received indirect WCF), and the control group (who received no feedback) was not compared in their study. Similarly, Mesfin [29] conducted a study on the effects of teacher versus guided self-correction on the grammatical accuracy of student-written texts. In light of these, it can be concluded that no research particularly investigated how university entrants' writing achievement is influenced by teachers' unfocused direct WCF accompanied by metalinguistic explanations and indirect WCF in the context of Ethiopian EFL learners.

Therefore, expecting conclusive and consistent results from studies with methodological flaws in their research designs is unrealistic. It is also highly dubious to conclude whether WCF is effective or ineffective or whether direct forms of WCF are more effective than indirect forms of WCF from research results that only focus on a small number of linguistic components like the aforementioned global studies. Since previous studies mainly relied on some predefined rule-based language features, these variations in their findings are attributed to methodological limitations in the provision of WCF [18,[24], [25], [26],^30^] or a lack of comparability and generalizability between these studies as a result of poor research design [31,32]. Furthermore, some studies lack a control group, although scholars generally agree that a control group should be included in experimental studies [17,[31], [32], [33], [34]]. It was also stated that “in an experimental study, the studies that lack a control group, one cannot tell if the improvements or effects found in the experiment group is because of the treatment until it is compared with the performance of a control group which received no treatment” as stated by Hudson & Liosa [[35], p. 85]. From this, it can be suggested that conducting additional studies in this field will undoubtedly be very important in improving our understanding of the issue and offering solutions to the issues faced by WCF practitioners. Therefore, the basic rationale for conducting this study was that there were no trustworthy, repeatable, broadly applicable research findings in the area of interest of the present study in the Ethiopian EFL context. Another justification for the current study's motivation was the lack of local studies that compare the effectiveness of direct WCF accompanied by a metalinguistic explanation with indirect WCF in improving learners' writing achievement. Thus, the current study was mainly initiated to examine the effectiveness of teachers' unfocused direct WCF, accompanied by a metalinguistic explanation and indirect WCFs, on enhancing the writing achievement of university entrants in the context of a university found in the northwestern part of Ethiopia, which was not the subject for other local studies in a similar concern like in the present study.

## 2 Review of literature

Writing is a useful language skill that university students need to develop and use in their daily lives. Its significance grows in writing in English, a language frequently utilized for intercultural communication and comprehension of necessary information [36]. Through writing, students can find contemporary methods to express their ideas and thoughts in a foreign language [3]. Nevertheless, writing is a very important language skill learning to write is a highly demanding language skill. For instance, Yea Yea, Othman and Wei [37] point out that learning to write is not an easy activity but rather a complicated talent that calls for rhetorical skills, as a textual understanding of the interaction between readers and authors is also crucial in developing academic writing abilities. Due to this, students have a variety of difficulties with English language writing competency because of its complexity, particularly when it comes to their writing abilities [38]. Due to these reasons, learners face numerous challenges in English language subjects, particularly in writing skills [39]. Consistent with this finding, results from local studies also confirmed that writing is the most challenging and last ability that students acquire [40]. As indicated by Dinsa [41] the main reason for Ethiopian EFL learners’ inadequate writing performance is ascribed to the writing skill involves much more than just using proper spelling, organizing ideas and words, and adhering to grammatical rules, this may be connected to writing difficulties. In a similar purpose, a rising number of earlier local studies stated that the target language grammatical errors are among the most impeding variables that hinder secondary school and university EFL students in Ethiopia from producing comprehensible academic texts as indicated in Abiy [42], Birhanu [43] and Dawit [44]. Although writing in English language is one of the most complex skills, since English is used worldwide as a lingua franca by people from various cultures, ethnic backgrounds, and social classes, learners must be equipped with the necessary knowledge of this skill, especially in English classrooms [45]. Therefore, to overcome these challenges, learners need to be supported in different ways; providing learners with written corrective feedback can be one of the ways that help them overcome the difficulties they face in their L2 writing classes.

### 2.1 Theoretical foundations for WCF in SLA

The role of feedback in language classrooms has undergone significant changes along with paradigm shifts in approaches and methodologies of second language instructions. These changes laid foundations supporting the role of WCF in SLA, despite disagreements among scholars regarding the roles and efficacies of different kinds of CF are still not settled. For example, as a result of the influence of theories of Behaviorism and Structuralism, CF was viewed as a crucial pedagogical tool in addressing EFL learners' linguistic weaknesses of EFL learners. Besides these, the role of CF is highly emphasized in the Noticing Hypothesis of Schmidt [46,47], in which acquiring a language requires intentional focus and knowledge of linguistic features. Also, Schmidt argued that by paying conscious attention to linguistic patterns and noticing unfavourable linguistic evidence, via WCF plays a key role in aiding learners in identifying the differences between their interlanguage – a natural language created by L2 learners – and the target language [48]). The role of CF was also underscored in Vygotsky's Sociocultural Theory [49,50], which contends that interaction between language learners and more knowledgeable other individuals who speak the target language more fluently mediates the gap and facilitates language learning. Thus, feedback matches the learners' zone of proximal development (ZPD) to enhance L2 learning, i.e., the area between their present and prospective proficiency levels [48,49]. Therefore, scaffolding like CF is used to gradually enhance the learner's comprehension skills and leads to independence in language learning.

The role of CF was also stressed in the Skill Acquisition Theory of DeKeyser [51], who confirmed that CF plays a role in enhancing learners’ declarative knowledge and turning it into procedural knowledge. Over time, the learner moves from controlled to automatic processing, which requires less focus, a faster rate of processing, and more accuracy. It offers clear knowledge and prevents inaccurate information from being formalized and carried out automatically as presented by Polio [52]. Therefore, DeKeyser [51] urges that more studies in the area are needed to determine the amount and type of helpful feedback during practice while highlighting the necessity of meaningful and adequate practice to attain automaticity. In general, different scholars like Ferris [53] recommend that L2 writers require feedback on their errors, direct instruction-focused training on various linguistic challenges, awareness of their linguistic inadequacies, and awareness of the need to improve their learning processes. In this regard, Sanz and Morgan-Short [54] pointed out that learners acquire rules or explicit evidence in the form of structural linguistics or CF. Based on these, it can be concluded that CF is a key pedagogical ingredient that helps learners improve their language learning.

### 2.2 Direct, indirect, and metalinguistic forms of WCF

Direct WCF is an input-providing strategy that directly offers a correct linguistic form nearby the error [20]. Indirect WCF, on the other hand, occurs if a teacher highlights or circles errors in students' papers without giving corrections [26]. Similarly, a metalinguistic explanation is another method of error correction in which error codes are written on the margins of the paper to inform students of the kinds of errors they have made or in which a teacher numbers the errors in the texts before providing grammatical explanations for each numbered error at the bottom of the page [17]. The indications can be exhibited in one of four ways: by underlining or circling the error, counting the errors in a line in the margin, or using a code to indicate both the fault's exact location and the type of errors that occurred in different forms of codes [17,55,56].

### 2.3 The role of written corrective feedback in SLA

However, Truscott [14] was a well-known scholar having the most extremist viewpoints on downplaying the role of WCF by commenting on the incapacity of teachers to correct errors in an organized and consistent approach, and the capacity and desire of students to pay attention to feedback which enabled him to draw his conclusions that the practice of CF is time-consuming and exhausting and that it interrupts learners' flow of information. For the same purpose, Truscott [14] linked WCF with issues such as pseudo-learning, learnability, and harmful side effects on the writing process; thus, he concluded that since it does not benefit L2 learners to progress in their writing performance, written corrective feedback should be abounded. In contrast, an increasing number of theories and empirical studies advocated the role of WCF in L2 classrooms. For instance, among theories that support for the role of written CF theoretical orientations, such as the interactionist hypothesis, Noticing Hypothesis, error analysis, and sociocultural theories, can be cited for their steadfast determination to establish that CF is an indispensable tool that plays a crucial role in facilitating L2 learners’ writing abilities. Besides these, the theoretical stance on the role of WCF in a plethora of empirical studies underscored the significance of WCF in L2 classrooms [[57], [58], [59], [60], [61]]; some of them have once again significantly been disputed [62].

However, WCF plays a key role in L2 writing classrooms, and the effectiveness of direct or indirect WCF in assisting L2 learners in enhancing their writing ability is still the source of disputes for scholars in the area of interest. For instance, Benson and DeKeyser [24] investigated the effects of WCF and language aptitude on tense verb accuracy and reported that both experimental groups outperformed their counterparts in the control groups on new pieces of writing, and these scholars concluded that both direct and indirect WCF are effective in helping learners writing improvement. Moreover, this was also discovered by other studies, such as Hosseniy; in which both intervention groups outperformed the control group in Hosseiny's [18] study on the effects of direct and indirect WCF on the usage of definite and indefinite articles. In a similar vein, a total of 21 papers on WCF were analyzed in a meta-analysis study carried out by Kang and Han [19]. The results indicated that no statistically significant differences between direct and indirect WCF provision strategies on learners' writing quality. Bitchener and Knoch [63] also conducted a study on the influence of WCF on linguistic development and found that direct WCF was more effective than the indirect form of WCF. Likewise, Eslami [23] indicated that learners in the experimental group who received indirect CF showed improvement more consistently in simple past tense usage than their counterpart learners in the direct CF who received direct error correction on their writings in a study of low-intermediate Iranian EFL students.

On the other hand, many scholars found that direct CF is superior to indirect CF. For instance, an increasing number of studies were reporting that direct written CF is the most effective provision strategy over its counterpart [26,27,63,64]. In proving this, for instance, WCF's influence on linguistic development. They found that direct written CF was more effective than the indirect form of WCF. Thus, it was observed that the results from the previous studies contradicted each other. Therefore, in the past, though several studies have extensively investigated the effects of direct and indirect WCF on students' writing proficiency, the findings are pretty contradictory [24,27,65], so no conclusive and consistent results were obtained.

In general, from the existing results in the field of study, it is challenging to draw conclusive and consistent conclusions due to the diversity of study focus, types of treatments, populations, and research designs [66]. Hence, even if there are ongoing debates regarding the efficacy of WCF, L2 learners want feedback on their writing and anticipate receiving it from their teachers; they want to know if the writing they have produced is appropriate or not based on the given feedback [31]. Likewise, instructors generally believe providing corrective error correction on learners' writing text is beneficial and essential for improving their writing. They think that WCF can significantly contribute to raising the level of writing correctness among their students as pointed out by Brown [67] and Hyland & Hyland [66], which influences instructors’ attitudes and beliefs, pedagogical implementation, and the offering of written CF that can benefit EFL learners most to improve their writing abilities. Thus, this is the main reason that the current researchers conducted this study in this area. Consequently, as these authors noted, rather than dismissing the importance of CF in learning second languages, the essential question that has to be pursued at this point is how to provide students with the best CF that can best benefit EFL learners to improve their writing ability. Thus, this was the underlying motivation that initiated the current researcher to conduct this study.

### 2.4 Feedback scope

The term the scope of feedback is used to refer to the number of errors to be corrected at a time. Under this consideration, we can have focused versus unfocused forms of corrective feedback provision techniques. Focused feedback comprises correcting predetermined language structures, whereas unfocused CF involves teachers giving WCF on a variety of erroneous language structures [68]. While focused WCF frequently contributes to studies that focus on assessing learners' grammatical accuracy, it is ineffective and unable to produce the required results in studies examining the influence of WCF on learners' overall writing achievement. However, several studies were conducted and offered empirical evidence advocating the effectiveness of focused/selective feedback over comprehensive feedback [[68], [69], [70]]; there are also doubts about the pedagogical and real-world applications of selective WCF in actual classrooms; thus, a growing number of scholars came in agreement with this stance and have questioned the ecological validity of targeted/focused feedback for second language classrooms if the ultimate goal of corrective feedback is to assist learners’ progress in overall accuracy rather than accuracy on predetermined linguistic forms, suggesting that a more thorough approach may be required [33,62,71].

On the other hand, Van Beuningen, De Jong, and Kuiken [27], maintained that writing assignments in selective CF studies resembled more grammar practices than authentic writing tasks, encouraging students to monitor the target feature deliberately. In a similar vein, studies by Ferris [62] and Hartshorn, Evans, Merrill, Sudweeks, Strong‐Krause and Anderson [72] showed that focusing exclusively on the acquisition/learning of particular linguistic forms falls short of meeting the prospects of learners and teachers in a natural class environment, where they anticipate feedback on all of their linguistic features in hopes of enhancing their written accurateness, and as a result, the findings of these studies lack reliability and empirical support. Similarly, Lee [73] stated that emphasizing exclusively on a small number of linguistic faults has minimal educational value. She also noted that these studies had been conducted in environments more akin to laboratories than in real classroom contexts, which could make it more challenging for learners to practice the lessons they acquired through feedback on new writing situations. It is also evident that from a practical standpoint, as stated by Ferris [62] and Storch [33], focusing only on particular error types might not be sufficient if a teacher's goal in correcting his students' written work is to improve accuracy in general, not just the use of one grammatical feature. To this end, it was also indicated that if “a teacher's purpose in correcting his or her students' written work is to improve accuracy in general, not just the usage of one grammatical feature,” unfocused CF is more in line with the widely accepted pedagogical practice [27, p. 6]. Consequently, correcting several errors can be more practical, ecologically valid, and beneficial for learners as indicated by Ferris [62] and Storch, [33]; this is because if learners receive some errors not corrected, they may perceive them as correct linguistic forms but they are not, so focused CF is not authentic in the actual classroom contexts. Likewise, students can find it confusing that some of their errors have been corrected while others have not, leading to the conclusion that if a study aims to improve learners' writing skills by emphasizing a small number of linguistic forms, this could be an unrealistic and unachievable dream.

In general, it can be stated that although WCF researchers provided extensive empirical evidence to shed light on the effectiveness of focused WCF, there is still a rising number of scholars voicing their apprehension towards this practice due to its lack of ecological validity [27,33,62,71,74]. From a practical perspective, L2 learners tend to commit various linguistic errors in writing, and the ultimate goal of teachers' providing WCF is to improve the overall writing accuracy rather than accuracy in specific linguistic categories [27]. Therefore, comprehensive WCF, which corresponds to the actual WCF practices in classroom settings, may deserve due attention in scholarship on writing [27,62,75,76]. Though to confirm whether this works in the Ethiopian context, studies that examine the effects of unfocused written corrective feedback on students' writing abilities are needed; thus, in response to these calls, the current study aimed to see the effects of unfocused direct written CF accompanied by metalinguistic explanations and indirect written CF on university entrant students’ writing achievement.

### 2.5 Feedback frequency

Feedback frequency refers to the amount of feedback given simultaneously in a single written document. Sustained and one-shot feedback provision strategies are the most common types that can be applied while providing WCF for L2 learners. According to Storch's [33] proposal, sustained WCF is a way of CF provision strategy where feedback is continually given on several writing texts. Therefore, to have fruitful changes in EFL learners' writing improvement, the researchers advised that a meaningful amount of information must be given to students for learning to occur, acknowledging educators' natural desire to fix everything [33]. Therefore, how educators would respond to the writing skills demands of their students, provide adequate feedback without changing everything, and do it in a time frame that motivates students to utilize and apply the material is still a dubious issue. As a skill is used more, it will become more accurate, according to the skill acquisition principle; practice leads to proceduralization, which leads to the automation of the skill [77]. Thus, in the current study, a sustained feedback provision strategy was used in which students in the experimental groups received written CF three times more than once to make sure that the observed changes in the post-test, if any, were continued further in another post-test and learners' improvements in their writing achievement was due to the intervention or not. Using a sustained feedback provision strategy also allows the researchers to consider the pedagogical implications and the applicability in broader circumstances as it gives confirmatory evidence that the observed changes were due to the treatment the study participant learners received in the experimental group.

## 3 Research questions

To achieve the main goal of this study, the research questions to be addressed in this study were.

1. Is there a statistically significant difference between the writing achievement mean scores of university students who are given direct WCF together with metalinguistic explanations and those who receive indirect WCF?
2. Is there a statistically significant difference between the writing achievement mean scores of university students' who receive direct WCF accompanied by metalinguistic explanations and those who are denied written corrective feedback?
3. Is there a statistically significant difference between the writing achievement mean scores of university students' who are provided with indirect written corrective feedback and those who are denied written corrective feedback?

## 4 Methods

### 4.1 Research design

This study used a quasi-experimental research design that employed paragraph writing tests as data collection instruments. A true experiment was not used because it is not recommended for human participants as far as moral, ethical, and practical related effects are concerned on human participants [78]. Thus, quasi-experimental research was preferable for the current study due to its advantages in an actual language classroom. Data were collected through two post-tests to determine the impact of the intervention on students' writing achievement. Three phases of the study typically comprise the pre-test, the first post-test and the second post-test design: (1) administering the pre-test that was used only as a covariate of the study and for providing feedback for study participants in the experimental groups, so after papers collected from the pre-test were commented and scored, this was followed with conducting the first post-test and measuring the dependent variable, (2) implementing the experimental treatment X on the participants and measuring the dependent variable, and (3) providing the second post-test, which once more measures the dependent variable of the study. When comparing the results of the first post-test and the second post-test, differences due to the application of the experimental treatment are then measured [78]. The current study lasted four weeks, during which the study participants in the intervention groups received a sustained unfocused WCF, and all study participant groups composed and submitted self-descriptive paragraphs three times.

### 4.2 Participants

The study participants were three intact classes of freshman students from a university located in northwest part of Ethiopia, during the first semester of the academic year of 2022-23.

### 4.3 Sampling strategy

However, truly randomized assignment is preferred over non-randomization in that it eliminates systematic differences that may already exist among groups as presented in Kirk [79] and Plonsky [80]; furthermore, Plonsky (80] pointed out that in applied linguistics research, random assignment is not always feasible due to practical or ethical considerations. Hence, instructional interventions in genuine learning contexts involving the utilization of intact classrooms are frequently sought by researchers studying the acquisition of second languages. Consequently, the threats to internal validity may be mitigated by the increased ecological validity provided by research in a setting that closely resembles real classroom settings where the results are used to make generalizations [81]. Hence, the researchers in this study used intact classes.

Concerning the inclusion of a control group, although using a control group is often advised, it is frequently impossible to do so in quasi-experimental research due to practical or ethical issues [80,82]. Thus, it is very important to keep in mind that, in some cases, using a comparison group makes sense. To this effect, as was already insisted by researchers, using intact classes is the most ecologically valid setting to conduct research when examining the effects of a specific instructional intervention [[80], [81], [82]]. It may be ideal in this situation to use a a comparison group (hereafter control group) group that is taking part in regular classroom teaching and used as the baseline reference for the experimental condition.

Having all these considerations in mind, the researchers in the current study used three intact classes, which means all of the students in each of the classes were allowed to be equally involved in the study process. As a result, using intact classes may give the researchers a chance to overcome the problems related to the group assignments. Therefore, in this study, three intact classes, i.e., two experimental groups and one control group, were participants in the study process. Therefore, the number of participants in this study was 135 generated from the direct group 44, in the indirect group 46, and 45 in the control group. It is very important to note that the participants in the control group were given compensation classes with similar materials that the study participants in the experimental groups were given during the intervention classes after data collection was completed.

### 4.4 Ethical considerations

Participants in this study were guaranteed confidentiality. The researchers confirmed to the study participants that there were no known potential risks associated with participation in this study. Pseudonyms were employed in the current study. The participants were instructed not to use their real names or any other kinds of personal identification in any portions of the paragraph since it was believed that doing so would allow them freedom and secrecy to engage in the study. Instead, they were told to use nicknames. In addition, the current study was examined and approved to achieve this goal by the Ethical Clearance Committee Board of Debre Tabor University (Reference No. SSH/06/2014). The participants in this study provided written consent before participating in the study process.

### 4.5 Variables of the study

Both dependent and independent variables were included in the current study. An independent variable is a variable that influences the dependent variable; it may also be referred to as a variable that produces a phenomenon, whereas the dependent variable is the variable that is impacted by the independent variable. As a result, the dependent variable in the current study was learners' writing achievement, which was supposed to be influenced by an independent variable, and the independent variable was written corrective feedback that was supposed to bring changes to learners’ writing achievement.

### 4.6 Instruments

In the current study, tests about self-descriptive paragraph writing were employed as a data collection tool. Using a self-descriptive paragraph was reasonable because writing descriptive paragraphs allows the study participant learners to write everything they know about themselves successfully. For that purpose, in the instructions that were provided for each participant in each of the testing sessions, it was stated to them what kind of information should be included in their paragraphs. Each study participant learner was asked to write a 150-word paragraph about him/herself within 40 min. Thus, controlled writing was the genre of writing used in the current study. The main argument in favour of using the control strategy in this study was that evaluating the participants’ written texts would be problematic if they were free to add anything to their paragraphs. For that reason, personalized and printed sheets of paper with directions and lists of points that the students expected to include in their paragraph writings were distributed to each of three self-descriptive paragraph writing test sessions, including the pre-test used for covariate of the study, the first post-test, and the second post-test.

#### 4.6.1 Reliability and validity of data collection instruments

##### 4.6.1.1 Construct validity

Construct validity and inter-rater reliability of the data-gathering test item were checked during the pilot study. In terms of validity, although a variety of validity can be checked, in this study, construct validity was checked. Construct validity can be understood as the ability of a test to accurately assess what it is intended to measure. With this regard, Heaton [83] stated that a test has construct validity if it can measure a specific quality in line with a theory of language behaviours and acquisition. Creswell [84] also added that construct validity is proven by examining if the results of an instrument are important, meaningful, useful, and have a purpose. As a result, it was determined that the testing item used in the current study has construct validity because it met all of the presumptions since it gave meaningful, useful, and purposeful results as intended.

##### 4.6.1.2 Inter-rater reliability

Reliability refers to the ability of a test to provide similar results used at different times. According to Drost [85], reliability refers to the ability of a measuring instrument to yield consistent results each time it is used as a measurement. According to Middleton [86], several types of reliability include test-retest, parallel forms, internal consistency, and inter-rater. However, in this study, only inter-rater reliability was used to check the instrument's reliability. To do that, during the pilot study, twenty papers were randomly taken and duplicated three times for three scorers. Based on the rubric developed, each scorer scored 20 papers. The intra-class correlation (ICC) of the items of the pre-test was calculated, and the statistical value of the ICC for the pre-test was found to be 0.963c, leading to the conclusion that the item has had acceptable inter-rater reliability. In line with this, scholars stated that the standard rule of thumb for ICC evaluation, is that a score of less than 0.40 is inadequate, a score of 0.40–0.59 is adequate, a score of 0.60–0.74 is acceptable, and a score of 0.75 and higher is outstanding as identified by Hallgren [87]. Hence, the intra-class correlation coefficient for this study was higher than 0.75; thus, it was determined that the data-collecting instrument has an acceptable level of inter-rater reliability.

### 4.7 Procedures

The participants and the classroom teacher were informed about the purpose of the study a week before the pre-test. Then, before the treatment, a pretest was administered to all participants of the study. After the participants in all groups completed and submitted their papers to the classroom teacher (experimenter), the experimenter collected learners' papers, provided WCF only for the study participant learners in the experimental groups, and marked all papers collected from all groups participating in the study. Then, the marked papers were photocopied, and the original papers were returned to the study participants so that they noticed the gaps in their writing skills based on the evidence given by written CF In contrast, the photocopied papers were submitted to the researchers for analysis. The main purpose of conducting the pre-test was to have data on the study participant learners’ language background that was used as a covariate for the study.

Similarly, as was in the pretest, in the first post-test, the participants were asked to write a comparable self-descriptive paragraph a week after they had received feedback on their pretest papers. As was previously said, the instructions, contents to be addressed, and steps to be undertaken for the first post-test were all comparable throughout the study process. Similarly, papers collected from the first post-test were commented on and photocopied similar to the way that was done in the pretest the original papers were given to the participants and the copies were used for analysis. In general, procedures similar to those used in the pretest were followed during the first post-test. Note that feedback was given only to study participants in both experimental groups.

The third writing test (post-test two) was identical in the genre of writing and the level of difficulty to the types of paragraphs learners wrote in the previous tests in the study. It was conducted two weeks after the first post-test was administered. As mentioned earlier, a similar procedure was followed for the second post-test. Finally, the experimenter provided feedback for learners in the experimental group and marked all papers from all groups; then, the papers were photocopied, and the copied papers were subsequently gathered and utilized for analysis. Furthermore, throughout the study, the participants were given guidance on how to structure a paragraph and what information to include in it. In this study, learners were given the same topic and instructions for paragraph writing throughout the study process. Besides these, learners in this test were given 45 min to complete a 150-word paragraph.

### 4.8 Data analysis

The researchers in the current study used a one-way (ANCOVA) data analysis technique to analyze the data. The reason for using a one-way ANCOVA was that the study involves one independent variable with different levels where two experimental groups including: direct written CF accompanied by metalinguistic explanations group and indirect written CF group in which these groups were given intervention and the control group was denied intervention from the experimenter. The second reason for using the analysis of covariance was to prevent the effects of other confounding variables, for instance, their linguistic background resources in the study process. The study also has one dependent variable, which allows for the analysis of changes in mean scores at different time points, such as pre-test, revision, first post-test, and second post-test, as well as investigates the variations in mean scores under various conditions, such as direct CF, indirect CF, and the control according to Roever & Phakiti [[88], p.154]. Therefore, a one-way ANCOVA was used to analyze the data from the tests to control for any possible variability in the scores of the three groups as presented in Pallant, [89]. In this study, throughout the analysis processes of the study, the participant groups’ pre-test writing mean score results of the pretest were used as covariates. This is because the covariate must be measured before the treatment or experimental manipulation is performed [90]. This scholar added that taking the covariate during the onset of the study is used to prevent scores on the covariate from also being influenced by the treatment. This technique is often used when evaluating the impact of an intervention or experimental manipulation while controlling for pre-test scores [90]. SPSS version 23 was employed to analyze the data.

## 5 Results

The study's main objective was to see the effects of unfocused direct written CF accompanied by metalinguistic explanations and indirect WCF, with an emphasis on both grammatical and non-grammatical language features on Freshman University students' writing achievement in the context of university found in the northwest part of Ethiopia. The results of the analysis of a one-way ANCOVA are presented below.

### 5.1 Comparing group results during the first post-test

Table 1 presents the results from descriptive statistics during the first post-test. When the results of the study participants in the experimental groups from the first post-test were compared with those of their counterparts in the control group obtained during the first post-test, it was clear that the writing means achievement scores of the study participants in the experimental showed significant changes. As can be seen in Table 1, there were substantial differences in the writing mean achievement scores between groups participating in the study in the first post-test. For example, study participants in the direct group who received direct written CF together with metalinguistic explanations scored a mean writing achievement score of 14.80 with a standard deviation of 3.695; study participants in the indirect group who received indirect written CF scored a mean writing achievement score of 12.15 with a standard deviation of 3.134; whereas, the study participants in the control group who denied written CF scored a mean writing achievement score of 9.80 with a standard deviation of 2.982. It is evident from this that study participant learners' writing performance varied significantly. Thus, after controlling the effects covariate, the study participant learners' writing mean achievement scores showed some significant differences during the first post-test, indicating that learners in all groups performed differently in their first post-test. For instance, when we compare the study participant learners' results of the direct group that received direct error correction with metalinguistic explanation with the control group who were denied feedback in this study and used as a baseline reference, learners in the experimental direct group outperformed their peers in the control group. As was shown in Table 1, learners in the direct written CF that received also outscored their counterparts in the indirect group who were given indirect written corrective feedback. In Table 1, it was also revealed that study participants in the indirect group who were given with indirect WCF performed better than their peers in the control group who did not receive feedback in the study. Therefore, from the results in Tables 1, it was observed that study participant learners in both experimental groups showed improvement when their results were compared with the results of the study participants in the control group. Since some variations are observed in learners' writing mean achievement scores, hence, the results revealing a difference in writing mean achievement score, and performing the tests between subjects' effects appeared necessary to determine whether or not the observed difference in learners’ writing mean achievement are statistically significant.

**Table 1:** Descriptive statistics showing mean score writing achievement results.

Dependent Variable: Writing Achievement Scores across Post-test1			
WCF	Post-test1 Writing Achievement Score		N
	Mean	Std. Deviation	
**DWCF with ME**	14.80	3.695	44
**IWCF**	12.15	3.134	46
**Comparison Group**	9.80	2.982	45
**Total**	12.23	3.840	135

**DWCF** = Direct Written Corrective Feedback.

**IWCF =** Indirect Written Corrective Feedback and.

**ME** = Metalinguistic Explanations.

From the results shown in Table 2, statistically significant differences between study participant groups were found. As was presented in the above table, the *p*-value for this analysis was found to be *F*(2,131) = 83.103, p = 0.001, p<α, α = 0.05, դp2> 0.559), providing evidence that the intervention given to research participants who were in the experimental groups resulted in statistically significant differences in their English language writing achievement scores. As shown in Table 2, the alpha [α] value, represented by the *p*-value, was found to be below its standard significant value level, which is α = 0.05, informing that there was a statistically salient difference in the study participant groups' writing performance in the first post-test. Thus, this result demonstrates that the intervention that the study participants received in the experimental groups helped learners participating in the study to enhance their writing performance. Moreover, the study's *eta* (effect size) is found to be = 0.559, which gives us a 56 % effect size, which is a large effect size, implying that the study had a strong effect size based on the benchmarks given by Cohen [91], who classified it as (Partial) **Eta Squared** as η^2^ = 0.01 a small **effect**; η^2^ = 0.06 a medium **effect**; η^2^ = 0.14 a significant **effect**. Thus, in light of these benchmarks, the current study had a large *eta* effect size, meaning a statistically salient relationship was found between independent and dependent variables in the first post-test; this means that the independent variable of the study significantly influenced the dependent variable of the study (Table 2). Thus, based on this, it can be said that WCF is very important instructional ingredient in helping university entrants improve their writing proficiency in Ethiopian EFL context at Debre Tabor University.

**Table 2:** Tests of between-subjects effects during post-test one.

Dependent Variable: Immediate Post-test1 Writing Achievement Score						
Source	Type III Sum of Squares	df	Mean Square	F	Sig.	Partial Eta Squared
Corrected Model	1616.573^a^	3	538.858	196.462	.000	.818
Intercept	49.122	1	49.122	17.909	.000	.120
Pre-test score	1060.985	1	1060.985	386.824	.000	.747
Conditions	455.872	2	227.936	83.103	.000	.559
Error	359.309	131	2.743			
Total	22167.000	135				
Corrected Total	1975.881	134				

a. R Squared = 0.818 (Adjusted R Squared = 0.814).

As shown in Table 3, the estimated marginal mean results were presented, and from the results, there was a change in the study participants' writing achievement mean scores compared with the original means in a similar test. For instance, the participants' unadjusted mean scores of the original mean in the first post-test were 14.80, 12.15, and 9.80 for the study participants in direct, indirect, and control groups, respectively, as presented in Table 1 above, however, the adjusted mean achievement writing score, as presented in Table 3, the direct group scored 14.456^a^, the indirect group scored 12.354^a^, and the control group scored 9.925^a^ showing that there is still a significant difference in the participants' writing performance after the effects of the pre-test scores were controlled as a covariate. Therefore, the results from the estimated marginal mean allow the researchers to better understand how the covariate has adjusted the original group mean scores (Table 3). Subsequently, a statistically significant difference in the participants' writing achievement was found, and running Bonferroni's pairwise comparisons was very important in identifying where the difference existed, a pairwise comparison is presented in the table below.

**Table 3:** Comparison of Original and Estimate Marginal Means during the Post-test one.

Dependent Variable: Immediate Post-test1 Writing Achievement Score				
	Mean	Std. Error	95 % Confidence Interval	
			Lower Bound	Upper Bound
Direct WCF with ME	14.456^a^	.250	13.961	14.951
Indirect WCF	12.354^a^	.244	11.871	12.838
Comparison Group	9.925^a^	.247	9.437	10.414

a. Covariates appearing in the model are evaluated at the following values: Pre-test Writing Achievement Score = 9.47.

A one-way ANCOVA for Bonferroni pairwise comparisons was computed, and the results are displayed in Table 4 to distinguish the difference in groups' writing achievement since statistically significant differences in writing performance across the groups were found in Table 3. From the comparison made in Table 4, the participant learners in the DG who were provided with direct WCF accompanied by metalinguistic explanations outperformed their counterpart peers study participants in both the IG who received indirect WCF and in the CG who received no feedback. When we compare the results of the study participant learners in the direct written CF group who were given direct written CF with metalinguistic explanations with the study participants’ writing performance of IG written CF those given with only indirect WCF, the results showed that study participants in the DG outperformed their peers in the IG with a mean score difference of 2.102* and with a *p*-value = 0.001. Similarly, the study participant learners in the DG outperformed their peers in CG with a mean score writing achievement difference of 4.531* with a *p*-value of 0.001, where in both cases, the *p***-**value was less than α = 0.05, that implies the participants in the DG outperformed the participants in both IG and CG in the first post-test. Therefore, based on the results shown in Tables 4 it can be concluded that first-year students who were provided with direct WCF accompanied by a metalinguistic explanation showed a significant improvement in their writing achievement when their results were compared with their counterpart peers inboth IG and CG.

**Table 4:** Bonferroni pairwise comparisons during post-test one.

Dependent Variable: Immediate Post-test1 Writing Achievement Score						
(I) written corrective feedback	(J) written corrective feedback	Mean Difference (I-J)	Std. Error	Sig.^b^	95 % Confidence Interval for Difference^b^	
					Lower Bound	Upper Bound
DWCF with ME	Indirect WCF	2.102*	.350	.000	1.252	2.951
	Comparison Group	4.531*	.352	.000	3.677	5.384
IWCF	Direct WCF with ME	−2.102*	.350	.000	−2.951	−1.252
	Comparison Group	2.429*	.347	.000	1.587	3.271
Comparison Group	Direct WCF with ME	−4.531*	.352	.000	−5.384	−3.677
	Indirect WCF	−2.429*	.347	.000	−3.271	−1.587

Based on estimated marginal means.

*. The mean difference is significant at the 0.05 level.

b. Adjustment for multiple comparisons: Bonferroni.

**IWCF** = Indirect Written Corrective Feedback.

DWCF = Direct Written Corrective Feedback.

**ME** = Metalinguistic Explanation.

Similarly, the participants in the IG outperformed study participant peers in the CG with a mean difference of 2.429* and with a *p*-value = 0.001, which is less than the alpha [α] standard value, which is α = 0.05, indicating that the study participant learners in the IG performed better in post-test one, and this implies that the changes observed in learners among learners in groups were due to the treatment they received. In light of these results, it can be inferred that both types of WCF are effective in assisting Ethiopian first-year university students; however, the extent of direct and indirect WCF assisted study participants' learners in improving their writing achievements was incomparable, so learners in the direct WCF group benefited more from the treatmentd more. From this, it can be concluded that providing learners with direct WCF with a metalinguistic explanation is specifically important when learners have limitations in linguistic background knowledge, as was the case with the current study's participants in Ethiopia. In this regard, Bitchener [92] contended that learners gain more from direct CF in a setting where they have had little exposure to the language. Moreover, Chandler [58] endorsed that offering direct WCF to EFL students who cannot recognize, differentiate, and correct their L2 errors extends their cognitive processing time and aids in the internalization of complicated grammatical structures. Thus, the current study's results go in line with Bitchener and Knoch [26], who postulated that direct WCF is beneficial because it gives students the first-hand experience necessary to evaluate hypotheses about the target language.

Moreover, from the findings of this study, it can be concluded that unfocused written corrective feedback is effective in assisting learners to enhance their writing achievement; thus, the results from this study support the arguments made by the scholars who underscored the ecological invalidity of studies that focused on a limited number of errors for the L2 classroom [33,62,71]. In line with this, Ferris [62] extends her stance by questioning whether the impact of highly targeted error categories could be extrapolated to improving students’ overall writing abilities in favour of unfocused WCF. This was also claimed in earlier studies, which commented that comprehensive CF is effective because it aids L2 students in becoming more aware of all the errors they have made and prevents them from building inaccurate linguistic structures in their interlanguage system [93]. It is also very detrimental that if learners received feedback on some limited linguistic errors while others were left uncorrected, learners could generalize that they are correct on the unfixed linguistic structures although they are not, which can lead them to fossilize the deviant language structures since they do not receive CF on them (Table 4).

### 5.2 Comparing group results during the second post-test

From the results in Table 5, study participants' mean score writing achievement showed significant variation across groups participating in the study. For instance, the study participant learners in the direct group's mean writing achievement was 16.86 with a standard deviation of 3.645, showing a noticeable change compared with the results of study participants in the indirect group whose mean score of writing achievement was 14.00 and with 3.419 standard deviation and control group whose mean writing achievement score which was mean of 9.96 and standard deviation of 3.96 that showed negligible changes compared with the study participant learners' mean writing achievement in the experimental groups. In addition, when we compare the results of the second post-test with the first post-test learners in the experimental groups, they showed significant improvements in their mean writing achievement score than those in the control group. To this effect, a one-way ANCOVA was carried out and displayed below to see if the observed changes in groups' mean achievement scores are statistically significant or not.

**Table 5:** Descriptive statistics showing mean score writing achievement results dependent variable: Writing achievement scores across Post-test2.

Post-test1 Writing Achievement Score			N
WCF	Mean	Std. Deviation	
**DWCF with ME**	16.86 14.00	3.645	44
**IWCF**		3.419	46
**Comparison Group**	9.96	3.960	45
**Total**	13.59	4.621	135

For the second post-test, a one-way ANCOVA was performed to determine whether the observed mean writing achievement score difference was considered to be statistically significant or not. Additionally, it was used to confirm that the improvements in the experimental groups' performance that were seen in the first post-test following their treatment continued in the second post-test, which was administered following another intervention on the paragraphs they had written for the first post-test. As depicted in Table 6, a statistically significant difference was found in the study participant students' writing achievement scores during the second post-test, confirming that learners' writing skills can be further enhanced if they are given continuous feedback. In this test, the *p*-value was found to be *F*(2,131) = 80.996, p = 0.001, p<α, α = 0.05, դp2> 0.553), which is less than the standard alpha value level that is 0.05, indicating that there was a statistically significant difference in the study participant groups' writing performance. In addition to this, the study's *eta* (effect size) was also found to be = 0.553, which gives us a 55 % effect size, implying that the study had a robust substantial effect size based on the benchmarks provided by Cohen [91], whose details are explained above. Thus, it can be inferred that there was a statistically meaningful improvement in the participants' writing achievement in the experimental groups who received feedback during the second post-test as was in the first post-test. Thus, the results in the second post-test were used to confirm the observed changes in the first post-test were due to the intervention the study participants received in the experimental groups. Consequently, in light of this, it can be concluded that if learners are provided with a sustained CF in their writing, they can enhance their writing performance until their writing texts become error-free. Therefore, conducting and further inspecting the unadjusted mean with the estimated mean can give us a further understanding of the changes observed in the analysis made in the second post-test.

**Table 6:** Comparing Between-Subjects Effects test scores during the Second Post-test.

Dependent Variable: Immediate Post-test 2 Writing Achievement Score						
Source	Type III Sum of Squares	Df	Mean Square	F	Sig.	Partial Eta Squared
Corrected Model	2093.042^a^	3	697.681	119.048	.000	.732
Intercept	141.180	1	141.180	24.090	.000	.155
Pre-test score	1019.365	1	1019.365	173.938	.000	.570
Conditions	949.355	2	474.677	80.996	.000	.553
Error	767.728	131	5.861			
Total	27776.000	135				
**Corrected Total**	**2860.770**	**134**				

a. R Squared = 0.732 (Adjusted R Squared = 0.725).

The unadjusted writing achievement mean scores for the direct, indirect, and control groups during the second post-test, as shown in Table 5, were 16.86, 14.00, and 9.96, respectively, demonstrating that there were still discernible differences in the study participant groups' writing achievement scores. In the same way, the study participant groups' unadjusted writing achievement mean scores for the direct group, indirect group, and control group also showed differences in groups as were revealed in Table 7, 16.531^a^, 14.198^a^, and10.078^a^, respectively, during the second post-test. The results presented in Table 7 suggested that there were still statistically significant differences in their writing performance. To find out where the difference was found, conducting Bonferroni's Pairwise Comparisons was found to be essential, and it was conducted and presented below.

**Table 7:** Estimated marginal means during Post-test Two.

Dependent Variable: Immediate Post-test2 Writing Achievement Score				
Written Corrective Feedback	Mean	Std. Error	95 % Confidence Interval	
			Lower Bound	Upper Bound
DWCF with ME	16.531^a^	.366	15.807	17.255
IWCF	14.198^a^	.357	13.492	14.905
Comparison Group	10.078^a^	.361	9.364	10.792

a. Covariates appearing in the model are evaluated at the following values: Pre-test Writing Achievement Score = 9.47.

As shown in Table 8, the results from Bonferroni's Pairwise Comparisons in the second post-test revealed that there were statistically significant variances in the study participating groups' writing performance in the direct WCF compared with the performance of the participants groups in the indirect and control groups. As demonstrated in Table 8, the study participants in the DG given direct WCF accompanied by metalinguistic outperformed their peers in the IG that received indirect WCF with a mean difference of 2.333* and with a *p*-value = 0.001, suggesting that there was a statistically noticeable difference in the participants' writing achievement in the direct and indirect groups. Similarly, the results showed that the participants in the DG outperformed their counterparts in the CG with a mean difference of 6.453* and *p*-value = 0.001, demonstrating that there was a meaningful difference in the writing achievement of the participants between those who received unfocused direct feedback with a metalinguistic explanation and those who did not receive feedback.

**Table 8:** Bonferroni Pairwise Comparisons (Post-hoc Analysis) during Post-test two.

Dependent Variable: Immediate Post-test2 Writing Achievement Score						
(I) written corrective feedback	(J) written corrective feedback	Mean Difference (I-J)	Std. Error	Sig.^b^	95 % Confidence Interval for Difference^b^	
					Lower Bound	Upper Bound
DWCF with ME	Indirect WCF	2.333*	.512	.000	1.091	3.574
	Comparison Group	6.453*	.514	.000	5.205	7.700
IWCF	Direct WCF with ME	−2.333*	.512	.000	−3.574	−1.091
	Comparison Group	4.120*	.508	.000	2.889	5.351
Comparison Group	Direct WCF with ME	−6.453*	.514	.000	−7.700	−5.205
	Indirect WCF	−4.120*	.508	.000	−5.351	−2.889

Based on estimated marginal means *. The mean difference is significant at the 0.05 level.

b. Adjustment for multiple comparisons: Bonferroni.

**DWCF** = Direct Written Corrective Feedback, **IWCF** =Indirect Written Corrective Feedback and **ME** = Metalinguistic Explanation.

Likewise, considerable statistical variations were found in the study participants' writing achievement between the learners in the indirect group who received treatment and the control group who were denied feedback. As displayed in Table 8, the participants in the IG outscored their counterparts in the control group with a mean score difference of 4.120*and with a *p*-value of 0.001, which is less than the standard value of alpha (α) that is 0.05, indicating that there was a statistically significant difference in study participants' writing achievement who received indirect WCF and those who did not. Thus, it can be inferred from this that comprehensive direct WCF together with metalinguistic explanations and indirect WCF are both effective pedagogical tools in enhancing Ethiopian first-year university students’ writing achievement; moreover, the findings indicated that direct WCF was superior to indirect WCF in helping learners improve their writing skills (Table 8).

Table 9 presents univariate tests from both post-tests. Based on the information presented in Tables 9, it can be concluded that written CF is an effective ingredient in EFL writing classes in the Ethiopian context. In both post-tests, statistically significant differences were found between groups’ writing achievement, as indicated in the analysis made above. For instance, in the first post-test the alpha [α] is given that *F*(2,131) = 83.103, p = 0.001, p<α, where α = 0.05, դp2> 0.559), and *F*(2,131) = 80.996, p = 0.001, p<α, where α = 0.05, դp2> 0.553) demonstrating that there was a statistically significant difference in the writing performance of the participants during the first post-test and the second post-test respectively with a strong eta effect size that can also be stated η, η2p > 0.553, and it gave us 56 % *eta* effect size in the first post-test and η, η2p > 0.553 yielded 55% *eta* effect size in the second post-test. These effect sizes indicate that there was substantially significant effect size value, which led to the conclusion that the relationship between variables was powerful during the first post-test. In general, it can be claimed that in the context of Ethiopian freshman university students, providing WCF plays an invaluable role in assisting them in enhancing their writing skills (Table 9).

**Table 9:** Univariate tests for both post-test one and two.

Dependent Variable: Immediate Post-test1&2 Writing Achievement Score												
Immediate Post-test1 Writing Achievement Score							Immediate Post-test2 Writing Achievement Score					
	Sum of Squares	Df	Mean Square	F	Sig.	Partial Eta Squared	Sum of Squares	df	Mean Square	F	Sig.	Partial Eta Squared
Contrast	455.872	2	227.936	83.103	.000	.559	949.355	2	474.677	80.996	.000	.553
Error	359.309	131	2.743				767.728	131	5.861			

The F tests the effect of written corrective feedback.

This test is based on the linearly independent pairwise comparisons among the estimated marginal means.

## 6 Discussion

The role of CF has been a contentious issue for an extended period in EFL research and SLA history. For instance, Truscott [14] was found to be a renowned and frontrunner scholar in having the most extremist viewpoints on any role WCF has in language classrooms. Other scholars in language instruction such as Kepner [94] and Sheppard [95] have the same viewpoints as Truscott on the role of WCF in second or foreign language learning. Although these scholars downplay the role of WCF in L2 classes, the results from the current study appeared to contradict their assumptions and demonstrate that WCF is a highly effective pedagogical tool that supports learners in identifying their interlanguage strengths and weaknesses, and based on the evidence they have via CF, they can overcome writing challenges they have in their target language performance. Thus, the current study's results align with study findings that found that WCF is a productive language instruction that facilitates EFL students [17,58,60,61]. Consequently, the current study's results found to substantiate the study results from scholars like Lee [16], who reached the consensus that corrective feedback is unquestionably necessary to facilitate L2 knowledge because mistakes and errors are expected at all stages of learning, despite shifting perceptions about what constitutes effective and meaningful feedback for students.

When it comes to the study's research questions, the first research question was (Is there a statistically significant difference found in students’ writing achievement between learners in the direct group provided direct WCF accompanied by metalinguistic explanations from those who received indirect written corrective feedback?) the results indicated a statistically significant difference between the study participants who received direct WCF accompanied by an explanation and those who were just given indirect written CF. As was presented above in Table 4, Table 8, learners in the direct group outscored their peers in the indirect group by a mean difference of 2.102* and with a *p*-value of 0.001 in the first post-test and with a mean difference of 2.333* and with a *p*-value of 0.001 in the second post-test demonstrating that learners in the direct accompanied by a metalinguistic explanations benefited more from the written corrective feedback that the experimental groups received in the study.

The second research question was about (Is there a statistically significant difference in learners' writing achievement between those in a direct group who received direct WCF accompanied by metalinguistic explanations and those in a control group who were denied corrective feedback on their written text?) The results showed that there was a statistically significant difference in the writing performance of the participant groups. For instance, the participants in the DG who were given direct correction accompanied by linguistic illustrations outperformed their peers in the CG who received no feedback with a mean score difference of 4.531* and with a *p*-value of 0.001 where the *p***-**value is less than α = 0.05, in the first post-test and with a mean difference of 6.453* and with a *p*-value = 0.00, in the second post-test, implying that the study participant learners in the DG those provided with direct WCF accompanied by metalinguistic explanations outperformed the study participants in the CG who received no feedback in the first post-test. Thus, from the results, it could be concluded that there were statistically significant differences between the participants who received direct WCF accompanied with a metalinguistic explanation and those who were denied feedback. This was also indicated in a study by Wondim et al. [28], who showed that learners in the direct WCF group performed better compared with other participant groups in their research; however, they failed to give a clear difference between study participant groups’ writing performance.

Regarding thethird research question, which was about (Is there any statistically significant difference in learners' writing achievement between students in the indirect group receiving indirect WCF and those in the control group who are denied corrective feedback?) The results indicated that a statistically significant difference was found between the participants who received indirect WCF and those in the control group who received no feedback. As presented in Table 8, the participants who received indirect WCF outperformed their counterparts in the CG who received no feedback on their writing achievement with a mean score difference of 2.429* and with a *p*-value = 0.001 in the first post-test, indicating that the participants in the IG performed better in the first post-test, and this implies that although indirect WCF was not as effective as direct WCF in helping L2 learners, it plays a key role in assisting learners to improve their writing skills. Similarly, in the second post-test, the participants in the indirect group who were provided with indirect WCF outperformed their peers in the control group who received no CF. The findings showed that there were statistically significant differences between the participants who received indirect CF and those who provided no feedback. In Table 8 the analysis of Bonferroni Pairwise Comparisons was presented. As presented in this table when the participants' mean writing achievement in the CG was subtracted from the participants ‘mean writing achievement of the IG, it yielded 4.120* indicating a noticeable difference in learners’ writing performance in these groups. This was also confirmed by a *p*-value = 0.001, which is less than the standard value of alpha (α) is 0.05, which confirms that statistically significant changes in the participants' writing achievement were found between those who received indirect WCF and those who did not receive it. In short, from the results of the study, it can be concluded that indirect WCF is an effective type of WCF that has the potential to help Ethiopian first-year university students; however, direct WCF found to be more effective than indirect WCF in helping Ethiopian university entrants improve their writing achievement.

However, the usefulness of WCF in enhancing students' writing correctness in second language learning has long been a topic of intense debate; for instance, some scholars have contended that WCF is useful for L2 writing practice [17,58,[60], [61], [62]], on the other hand, others disagree with this stance and argue that it should not be used in L2 classrooms [14,34,94]. The results of the current study appeared in supporting those works that believe that WCF plays a crucial role in helping students improve their writing abilities. Therefore, based on a cognitive and psycholinguistic framework in SLA, the focus appears to be shifting toward written corrective feedback's potential to support learners' interlanguage development and determine whether L2 learning is possible when written corrective feedback is presented and acted upon [26,61,68]. Thus, the results from the current study provide further empirical evidence in support of the efficacy of WCF, and it also adds some convincing knowledge to the literature on the effectiveness of different types of WCF. Therefore, these results substantiate the results of earlier studies that found that direct WCF is more effective in assisting students with lower linguistic proficiency levels since they have a relatively inadequate linguistic background as indicated by Bakri [96] and Bitchener [92].

Additionally, this study refutes the Truscott-proposed avoidance phenomena using the analyses of Van Beuningen et al.’s [27] study, whose findings suggest that comprehensive CF is a useful educational tool that teachers can use to help L2 learners improve their written accuracy over time consistent with the results of the current study. Furthermore, the study's results agree with those of earlier studies that advocate the effectiveness of direct WCF in helping L2 learners enhance their writing quality. In line with this, scholars contended that the learners receiving direct WCF could instantaneously internalize and be familiar with correction and the form; conversely, those whose texts were revised via indirect CF were incapable of doing so since they did not know whether their own hypothesized correction was indeed accurate [97].

## 7 Implications

From the results of the study, the following implications were forwarded. Thus, in light of the study's results, it was observed that WCF is a tremendously helpful pedagogical tool in helping first-year university students improve their writing performance. Consequently, it was concluded WCF can play a pivotal role in assisting to enhance Ethiopian EFL learners to be successful students in their target language writing abilities and their SLA, which is directly linked with their academic achievement in general. To this end, scholars opined that writing is such an essential part of learning English as a foreign language (EFL) that it is necessary to help students succeed academically as presented in Aliyu [98]. As a result, one of the most important indicators of undergraduate students' academic performance is their writing ability [99,100]. Therefore, this skill helps learners in succeeding in their SLA in particular and academic achievement in general. After all, writing is one of the macro skills that helps learners build their grammar and enrich their vocabulary, which can help them succeed in other content subjects. In line with this, it was stated that writing is a skill that plays a crucial role in educational settings as the most used mode of communication between teachers and students next to oral communication [28].

Therefore, to ensure improvements in writing skills, this skills must be assessed at each step of writing. Consequently, it is essential to continuously and formatively provide comments and feedback on writing tasks to strengthen writing skills [101]. For writers to become proficient in writing in their first language (L1), second language (L2), and especially foreign language (FL), it is crucial to offer them ongoing, formative feedback. Therefore, the principal objective of this study was to assist students in becoming effective and efficient writers who could easily, effortlessly, and convincingly articulate their ideas. It is also evident that the results have some pedagogical implications because one of the primary objectives of teaching foreign languages is to build communicative competence and to provide students with the ability to use languages for communication [102]. Therefore, to allow students of various levels to utilize the target language structures effectively, it is necessary to implement well-designed teaching processes, actively involve instructors and students, provide useful feedback in various ways, and employ effective teaching approaches [103]. In light of this, the researchers in the current study advise practitioners to utilize WCF by taking into account the important pedagogical concerns related to the delivery of WCF, such as what, when, where, and how of delivering and how often it should be given. This was also a concern of earlier studies that investigated questions concerning the reasons for correcting errors, which errors should be corrected, when (in which phase of writing), and how and who should correct them [104].

Therefore, the current study's findings have consequential effects in practice for SLA since L2 teachers can utilize extensive error corrections in their writing classes to enhance their students' writing performance in creating L2 material at the level they anticipate from their learners. Even though this kind of feedback entails identifying and fixing all grammatical and non-grammatical errors in the students' writing, it was more surprising to find that giving learners direct corrections of the errors with metalinguistic explanations – giving brief rules or notes to explain the reasons for the corrections was found to be the most effective method for assisting students in enhancing their written text. Therefore, by providing this kind of feedback, L2 teachers can assist their students in precluding errors beingfossilized and improving their writing abilities.

## 8 Limitations and recommendations of the study

The study did not consider any other kinds of feedback; it merely focused on two types of feedback: direct WCF accompanied by metalinguistic explanations and indirect WCF; therefore, it is uncertain how efficient this feedback is compared to other forms of feedback.

The study only assessed the participants' ability to write paragraphs while creating L2 content right after getting comments. It is unknown if the feedback's effects will remain or be transferrable to other writing assignments once a considerable amount of time has passed after the learners have got it.

In this study, only the unfocused WCF provision strategy gained emphasis, so it is unknown if the comparison of an unfocused approach with a focused WCF approach is considered.

The study did not consider methodological, learner, and context variables in its investigations, so it is unknown if the study was conducted by considering these variables in its design.

The study only focused on teacher-initiated feedback; therefore, it excluded peer feedback and self-feedback on learners’ writing capabilities; thus, further studies are recommended to include these variables in their investigations.

Therefore, in light of the previously mentioned limitations of the study, additional studies are needed in the future on the following few potential new developments.

F095 Above, it was made clear that this study only looked at the effects of direct WCF with metalinguistic explanations and indirect WCF on students' writing achievement; consequently, it is suggested that future researchers consider different types of written CF such as peer and self-correction as doing so may help them gain a better understanding of these areas of concern.
F095 Future studies are advised to examine the impacts of focused and unfocused WCF strategies on learners' writing to have a better understanding of how WCF affects students' writing skills.
F095 It is recommended that future researchers take into account the influence of methodological, learner, and contextual variables on the findings of their study when doing a study on a comparable topic.
F095 We also recommend investigating the long-term and transfer effects of feedback long-term and transfer effects on L2 writing abilities to provide more knowledge in these areas of concern.
F095 It is also suggested that future researchers investigate the use of technology-based feedback tools, such as automatic error-correction software or online writing coaches, to give L2 learners more efficient and customized feedback for each individual.

## 9 Conclusions

The study's results clearly show that WCF is a viable tool that assists university entrants in learning to write and enhance their writing abilities in English language. Therefore, the study's results have bolstered the knowledge in the area of WCF in improving learners' writing performance in Ethiopian EFL contexts, specifically in first-year university students' English language writing achievement. Based on the study's results, it can be concluded that both direct and indirect forms of WCF can highly influence Ethiopian university entrants' writing a. Moreover, the study's findings demonstrate that participants in the direct group who received direct WCF accompanied by metalinguistic explanations of the corrected errors outperformed those participants in the indirect group who received only indirect WCF and the participants in the control group who were denied feedback; thus, the results are informative that if EFL learners in Ethiopia context are given with direct WCF accompanied by metalinguistic explanations can be benefited the most from the WCF in enhancing their writing abilities. As a result, the study shows that providing explicit WCF and supplementing the corrections with some explanations is much better than using alternative CF provisions in EFL circumstances when learners lack certain crucial language resources.

Additionally, it was noted from the study's findings that students in the indirect group who were only given indirect WCF (underlining or circling) ill-formed linguistic structures outperformed their counterparts in the control group who were denied WCF on their paragraph writing. This might be interpreted as supporting the claim given above that learners in the direct and indirect experimental groups benefitted more from written corrective feedback. In general, it was concluded that WCF plays a facilitative role in enhancing EFL students' writing achievement. Therefore, it is reasonable to conclude that university EFL instructors can employ unfocused written corrective feedback while editing students' written work. Moreover, they can also use both direct and indirect WCF; however, if they want to aid their students more, they are encouraged to use direct WCF accompanied by metalinguistic explanations where students can receive all correctable errors and justifications about the corrections methods are supplemented.

## Funding

This research did not receive specific grants from public, commercial, or not-for-profit funding agencies.

## Data availability statement

No data used for this study has been associated with or deposited into a publicly available repository; as a result, the data in this study were exclusively used in this study. Finally, the data will be made available on any request.

## Approval ethical committee

Every study involving human participants has to be evaluated by the Ethical Clearance Committee Board before it is implemented in any study. To that aim, the current study was examined and approved to achieve this goal by the Ethical Clearance Committee Board of Debre Tabor University (Reference No. SSH/06/2014). The participants in this study provided written, full consent before participating.

## Additional information

No additional information is available for this paper.

## CRediT authorship contribution statement

**Baymot Mekuriaw Wondim:** Writing – review & editing, Writing – original draft, Validation, Software, Methodology, Investigation, Formal analysis, Data curation, Conceptualization. **Kassie Shifere Bishaw:** Writing – review & editing, Supervision, Conceptualization. **Yinager Teklesellassie Zeleke:** Writing – review & editing, Supervision, Conceptualization.

## Declaration of competing interest

The authors declare that they have no known competing financial interests or personal relationships that could have appeared to influence the work reported in this paper.

## References

[1] Klimova B. (2012). The importance of writing. Paripex - Indian J. Res..

[2] Junianti R., Pratolo B.W., Wulandari A.T. (2020). The strategies of learning writing used by EFL learners at a higher education institution. Ethical Lingua: Journal of Language Teaching and Literature.

[3] Rao P.S. (2017). The characteristics of effective writing skills in English language teaching. Research Journal of English.

[4] Mitchell M., K (2018). Constructing writing practices in nursing. J. Nurs. Educ..

[5] Özdemir E., Aydın S. (2015). The effects of blogging on EFL writing achievement. Procedia-social and behavioral sciences.

[6] Mahmoudi A. (2017). Effect of planning on Iranian intermediate EFL learners' mastery of writing skills. TPLS.

[7] Mulugeta F. (2021). Addis Ababa University students’ strategy use in a reading-to-writing Task. The Ethiopian Journal of Education.

[8] Surur A.N., Dengela S.T. (2019). The relationship between Writing Anxiety and Students’ Writing Performance at Wolkite University First Year English Major Students. International Journal of English Literature and Social Sciences.

[9] Han J., Hiver P. (2018). Genre-based L2 writing instruction and writing-specific psychological factors: the dynamics of change. J. Sec Lang. Writ..

[10] Chomsky N. (1959). On certain formal properties of grammar. Inf. Control.

[11] Corder S.P. (1967). The significance of learner's errors. Int. Rev. Appl. Ling.: Int. Rev. Appl. Ling..

[12] Phuket P., Othman N. (2015). Understanding EFL students' errors in writing. J. Educ. Pract..

[13] Sermsook K., Liamnimitr J., Pochakorn R. (2017). The impact of teacher corrective feedback on EFL student writers' grammatical improvement. Engl. Lang. Teach..

[14] Truscott J. (1996). The case against grammar correction in l2 writing classes. Lang. Learn..

[15] Erdogan V. (2005). Contribution of error analysis to foreign language teaching. Mersin University Journal of the Faculty of Education.

[16] Lee I. (2017).

[17] Bitchener J. (2008). Evidence in support of written corrective feedback. J. Sec Lang. Writ..

[18] Hosseiny M. (2014). The role of direct and indirect written corrective feedback in improving Iranian EFL students' writing skills. Procedia-Social and Behavioral Sciences.

[19] Kang E., Han Z. (2015). The efficacy of written corrective feedback in improving L2 written accuracy: a meta-analysis. Mod. Lang. J..

[20] Bitchener J., Storch N. (2016). Written corrective feedback for L2 development. In *written corrective feedback for l2 development*. Multilingual Matters. Iran. J. Lang. Teach. Res..

[21] Papi M., Bondarenko A., Wawire D., Jiang C., Zhou C. (2020). Feedback-seeking behaviour in second language writing: motivational mechanisms. Read. Writ..

[22] Diab N. (2016). A comparison of peer, teacher and self-feedback on the reduction of language errors in student essays. System.

[23] Eslami E. (2014). The effects of direct and indirect corrective feedback techniques on EFL students' writing. Procedia-Social and Behavioral Sciences.

[24] Benson S., DeKeyser R. (2019). Effects of written corrective feedback and language aptitude on verb tense accuracy. Lang. Teach. Res..

[25] Westmacott A. (2017). Direct vs. indirect written corrective feedback: student perceptions. Íkala, Rev. Leng. Cult..

[26] Bitchener J., Knoch U. (2010). Raising the linguistic accuracy level of advanced L2 writers with written corrective feedback. JSLW.

[27] Van Beuningen C., De Jong N., Kuiken F. (2012). Evidence on the effectiveness of comprehensive error correction in second language writing. JLL.

[28] Wondim B.M., Bishaw K.S., Zeleke Y.T. (2023). Effects of teachers' written corrective feedback on the writing achievement of First-year Ethiopian university students. Educ. Res. Int..

[29] Mesfin M. (2020). The effects of teacher versus guided self-correction on the grammatical accuracy of student-written text: university EFL Students in Focus. Int. J. Curr.Res.Aca.Rev..

[30] Shintani N., Ellis R., Suzuki W. (2014). Effects of written feedback and revision on learners' accuracy in using two English grammatical structures. LL.

[31] Ferris D. (2004). The “grammar correction” debate in L2 writing: where are we, and where do we go from here? (And what do we do in the meantime…?). JSLW.

[32] Guenette D. (2007). Is feedback pedagogically correct? Research design issues in studies of feedback on writing. J. Sec Lang. Writ..

[33] Storch N. (2010). Critical feedback on written corrective feedback research. ILES.

[34] Truscott J. (2007). The effect of error correction on learners' ability to write accurately. JSLW.

[35] Hudson T., Llosa L. (2015). Design issues and inference in experimental L2 research. Language Learning 65.

[36] Fareed M., Ashraf A., Bilal M. (2016). ESL learners' writing skills: problems, factors and suggestions. JESS.

[37] Yea L., Othman J., Wei L. (2020). The use of metadiscourse in academic writing by Malaysian first-year ESL doctoral students. IJA (Int. J. Acarol.).

[38] Alvi S., Haider K., Aziz F., Rehman N.A. (2020). English 5 History of educational policy-making and planning in Pakistan. The International Research Journal of Usooluddin.

[39] Ali A., Javed M., Shabbir G. (2017). Assessing ESL students' literal, reorganization, and inferential reading comprehension abilities. JER.

[40] Beleta D.M., Kebede S. (2022). Writing investigating the correlation of EFL students writing apprehension and writing performance: dire Dawa University in focus, Ethiopia. Journal of Languages, Linguistics and Literary Studies.

[41] Dinsa M.T. (2023). EFL students' writing strategies use in Ethiopia: gender and year level. Cogent Education.

[42] Abiy Y. (2013). Students' first language writing skills and their English language proficiency as predictors of their English language writing performance. J. Lang. Cult..

[43] Birhanu S. (2013). Assessing pre-engineering students' writing errors at Bahir Dar University, Ethiopia. J. Media Commun. Stud..

[44] Dawit A. (2014). The effect of communicative grammar on journalism students‟ writing skills. Academic Journals in Journal of Media and Communication Studies.

[45] Dewi A. (2015).

[46] Schmidt R., R (1990). The role of consciousness in second language learning. Appl. Linguist..

[47] Schmidt R., Robinson P.J. (2001). Attention. In cognition and second language instruction. Camb. Times: Cambridge University Press.

[48] Schmidt R. (2012). Attention, awareness, and individual differences in language learning. Perspectives on individual characteristics and foreign language education.

[49] Vygotsky L., Mind L. (1978).

[50] Vygotsky L., Wertsch J.V. (1981). The Concept of Activity in Soviet Psychology.

[51] DeKeyser R., Van Patten B., Williams J. (2007). Theories in Second Language Acquisition: an Introduction.

[52] Polio C. (2012). The relevance of second language acquisition theory to the written error correction debate. J. Sec Lang. Writ..

[53] Ferris D.R. (2002).

[54] Sanz C., Morgan‐Short K. (2004). Positive evidence versus explicit rule presentation and explicit negative feedback: a computer‐assisted study.

[55] Ellis R. (2009). A typology of written corrective feedback types. ELT J..

[56] Ferris D., Roberts B. (2001). Error feedback in the l2 writing classes: how explicit does it need to be?. J. Sec Lang. Writ..

[57] Bitchener J., Knoch U., U (2008). The value of written corrective feedback for migrant and international students. LTReport.

[58] Chandler J. (2003). The efficacy of various kinds of error feedback for improvement in the accuracy and fluency of L2 student writing. JSLW.

[59] Ellis N. (2005). At the interface: dynamic interactions of explicit and implicit language knowledge. Stud. Sec. Lang. Acquis..

[60] Ferris D. (1999). The case for grammar correction in L2 writing classes: a response to Truscott (1996). J. Sec Lang. Writ..

[61] Sheen Y. (2007). The effect of focused written corrective feedback and language aptitude on ESL learners' acquisition of articles. Tesol Q..

[62] Ferris D. (2010). Second language writing research and written corrective feedback in SLA: intersections and practical applications. Studies in SLA.

[63] Bitchener J., Knoch U. (2009). The relative effectiveness of different types of direct written corrective feedback. System.

[64] Bitchener I., Young S., Cameron D. (2005). The effect of different types of corrective feedback on ESL student writing. J. Sec Lang. Writ..

[65] Shintani N., Ellis R. (2013). The comparative effect of direct written corrective feedback and metalinguistic explanation on learners' explicit and implicit knowledge of the English indefinite article. J. Sec Lang. Writ..

[66] Hyland K., Hyland F. (2006). Feedback on second language students' writing. Lang. Teach..

[67] Brown J. (2007). Feedback: the student perspective. Res. Post-Compulsory Educ..

[68] Ellis R., Sheen Y., Murakami M., Takashima H. (2008). The effects of focused and unfocused written corrective feedback in an English as a foreign language context. System.

[69] Sheen Y., Wright D., Moldawa A. (2009). Differential effects of focused and unfocused written correction on the accurate use of grammatical forms by adult ESL learners. System.

[70] Sun S. (2013).

[71] Liu Q., Brown D. (2015). Methodological synthesis of research on the effectiveness of corrective feedback in L2 writing. J. Sec Lang. Writ..

[72] Hartshorn K.J., Evans N.W., Merrill P.F., Sudweeks R.R., Strong‐Krause D.I., Anderson N.J. (2010). Effects of dynamic corrective feedback on ESL writing accuracy. Tesol Q..

[73] Lee I. (2018). Teachers' frequently asked questions about focused written corrective feedback. TESOL J..

[74] Karim K., Nassaji H. (2020). The revision and transfer effects of direct and indirect comprehensive corrective feedback on ESL students' writing. Lang. Teach. Res..

[75] Bonilla López M., Van Steendam E., Buyse K., Speelman D. (2021). Comprehensive corrective feedback in foreign language writing: the response of individual error categories. JWR.

[76] Van Beuningen C. (2010). Corrective feedback in L2 writing: theoretical perspectives, empirical insights, and future directions. Int. J. Engl. Stud..

[77] DeKeyser R. (2020). Theories in Second Language Acquisition.

[78] Ary D., Jacobs L., Sorensen C. (2010).

[79] Kirk R., Millsap R.E., Maydeu-Olivares A. (2009). The Sage Handbook of Quantitative Methods in Psychology.

[80] Plonsky L., Loewen S., Sato M. (2017). Routledge Handbook of Instructed Second Language Acquisition.

[81] Mackey A. (2017). The Routledge Handbook of Instructed Second Language Acquisition.

[82] Mackey A., Gass S.M. (2015).

[83] Heaton J. (1988).

[84] Creswell J.W. (2020).

[85] Drost E. (2011). Validity and reliability in social science research. Educ. Res. Perspect..

[86] Middleton F. (2019). https://www.scribbr.com/methodology/reliability-vs-validity/.

[87] Hallgren K. (2012). Computing inter-rater reliability for observational data: an overview and tutorial. Tutorials in Quantitative Methods for Psychology.

[88] Roever C., Phakiti A. (2017). *Quantitative methods for second language research: A problem-solving approach*.

[89] Pallant J. (2010).

[90] Pallant J. (2016).

[91] Cohen J. (1988).

[92] Bitchener J. (2012). A reflection on ‘the language learning potentials of written CF. JSLW.

[93] Lalande J. (1982). Reducing composition errors: an experiment. TMLJ.

[94] Kepner C. (1991). An experiment in the relationship of types of written feedback to the development of second-language writing skills. MLJ.

[95] Sheppard K. (1992). Two feedback types: do they make a difference?. RELC.

[96] Bakri H. (2016). The role of individual differences in second language writing corrective feedback. AWEJ.

[97] Van Beuningen C.V., Jong N.D., Kuiken F. (2008). The effect of direct and indirect corrective feedback on L2 learners’ written accuracy. Int. J. Appl. Ling..

[98] Aliyu M.M. (2020). Exploring the nature of undergraduates' peer collaboration in a PBL writing process. International Journal of Language Education.

[99] Ferris D. (2007). Preparing teachers to respond to student writing. J. Sec Lang. Writ..

[100] Graham C.R. (2006).

[101] Hyland K. (2019).

[102] Spada N. (2010). Beyond form-focused instruction: reflections on past, present and future research.

[103] Pawlak M. (2014).

[104] Bitchener J., Ferris D.R. (2012).

